# Movement Synchrony Forges Social Bonds across Group Divides

**DOI:** 10.3389/fpsyg.2016.00782

**Published:** 2016-05-27

**Authors:** Bahar Tunçgenç, Emma Cohen

**Affiliations:** Institute of Cognitive and Evolutionary Anthropology, School of Anthropology and Museum Ethnography, University of OxfordOxford, UK

**Keywords:** children, in-group attitudes, minimal group paradigm, out-group attitudes, prosociality, social bonding, cooperation, movement synchrony

## Abstract

Group dynamics play an important role in the social interactions of both children and adults. A large amount of research has shown that merely being allocated to arbitrarily defined groups can evoke disproportionately positive attitudes toward one's in-group and negative attitudes toward out-groups, and that these biases emerge in early childhood. This prompts important empirical questions with far-reaching theoretical and applied significance. How robust are these inter-group biases? Can biases be mitigated by behaviors known to bond individuals and groups together? How can bonds be forged across existing group divides? To explore these questions, we examined the bonding effects of interpersonal synchrony on minimally constructed groups in a controlled experiment. In-group and out-group bonding were assessed using questionnaires administered before and after a task in which groups performed movements either synchronously or non-synchronously in a between-participants design. We also developed an implicit behavioral measure, the Island Game, in which physical proximity was used as an indirect measure of interpersonal closeness. Self-report and behavioral measures showed increased bonding between groups after synchronous movement. Bonding with the out-group was significantly higher in the condition in which movements were performed synchronously than when movements were performed non-synchronously between groups. The findings are discussed in terms of their importance for the developmental social psychology of group dynamics as well as their implications for applied intervention programs.

## Introduction

Peer relationships among friends are at the core of children's lives. Cooperative bonds define children's social sphere of activity, guiding decisions about whom to interact with and whom to avoid. Research has established the relevance of group markers, such as language, skin color and age, in guiding affiliative and cooperative social preferences (see Kinzler et al., 2010). While this ‘groupishness’ typically engenders positive prosocial sentiment and behavior toward friends in one's own group, group-level preferences can also entrench cross-group divides, ultimately precipitating anti-social prejudice and injustice (e.g., Tajfel, 1970). Although much is now known about how these lines get drawn, there has been relatively little investigation into how they can be effectively erased. Here we investigate the effect of movement synchrony, a core element of interpersonal behavior in social play, conversation, music, sport and exercise on group-based social bonds and divides. Synchrony has positive effects on social bonding and cooperation in adults (e.g., Wiltermuth and Heath, 2009) and children (e.g., Cirelli et al., 2014a; Rabinowitch and Knafo-Noam, 2015; Tarr et al., 2015). Successful social and educational programs frequently incorporate sports, music and movement activities to improve relationships within and across groups (Bailey, 2005; Schellenberg et al., 2015). Yet, the causal mechanisms underlying their success are largely unknown. The current study explores the idea that performing synchronous movements reduces negative attitudes toward out-groups and bonds individuals across existing group divides.

A vast amount of research on adults suggests that group identities can bond people together and that group-based preferences powerfully shape social attitudes and behaviors toward others (e.g., Tajfel, 1970). People respond more positively to in-group than out-group members (Hewstone et al., 2002), favor their own group and discriminate against the out-group (for a review, see Fiske, 2002), and empathize with and help in-group members more than out-group members (e.g., Stürmer et al., 2005, 2006). This stark distinction in attitudes toward in-group vs. out-group members is known as in-group favoritism and out-group bias (Tajfel and Turner, 1979), or groupishness. In-group favoritism refers to the attribution of positive qualities to one's own group and preferential cooperation toward in-group members, while out-group bias refers to negative attributions and discrimination against the out-group. Despite its potential negative implications for social discrimination, groupishness may be viewed as a kind of cognitive shortcut that can help make sense of the complex social world via generalizing rules of thumb (Tajfel, 1970). In this regard, skills enabling group-based perception and categorization are crucial for social development.

Representation and categorization of the social world along group lines as “us” vs. “them” emerges early in childhood, and potentially has foundations in infancy. Infants as young as 3 months old better recognize and prefer looking at faces of the race they see most often to faces of an unfamiliar race (Kelly et al., 2005). Between 6 and 12 months of age, infants start displaying age-based preferences; they listen longer to the sounds of their peers (Legerstee et al., 1998) and also look longer at the images of their peers (Sanefuji et al., 2006) over adults. Starting from 5 months, infants prefer to look at adults who speak their native language over those who are foreign speakers (Kinzler et al., 2007) and they also prefer adults who speak with a native accent over those who speak with a foreign accent (Kinzler et al., 2007). Ten-month-old infants prefer to receive toys from an adult who speaks their native language (Kinzler et al., 2007) and 14-month-old infants preferentially imitate a native speaker over a foreign speaker (Buttelmann et al., 2013).

In early childhood, group-based social categorization continues to shape children's social attitudes and behaviors, including affiliation. For instance, 3-year old children more readily select objects and activities endorsed by same-sex and same-aged others (Shutts et al., 2010). When asked who they would like to be friends with, 5-year-old children choose native language speakers over foreign language and foreign-accented speakers (Kinzler et al., 2007, 2009). Gender also becomes important in guiding children's friend choices around 4–6 years of age (Martin et al., 1999). Importantly, by this age, group-related biases appear to acquire a conventional element; children not only assume that others would also prefer same-sex partners, but they also anticipate more social approval from others if they play with same-sex peers (Martin et al., 1999). Indeed, this preference for same-sex peers increases until adolescence, when an interest for opposite-sex partners starts to reverse the pattern (Ruble et al., 2006). Notably, however, race-based preferences do not reliably exist until around 4–5 years of age (Bennett and Sani, 2003; Shutts et al., 2010; Weisman et al., 2014; though see Dunham et al., 2013). There is also evidence that, from the age of 5, children's ethnicity-based group biases cut across minimal group preferences. They report liking a member of an out-group more if the out-group is of the same-ethnicity than if he or she is of different ethnicity to them (Nesdale et al., 2004). Further, social status becomes increasingly important: children report liking an out-group more if the out-group has a high status than a low status, and may even prefer to switch their group membership (Nesdale et al., 2004).

Group-related biases emerge even in the absence of real-world divides to which children may be regularly exposed through their development, such as sex, race, age, or language differences. Research within the “minimal group paradigm” has demonstrated that, in both adults and children, arbitrary group memberships can be sufficient to induce group-related stereotyping and preferences (Tajfel et al., 1971). In a minimal group paradigm, people are randomly assigned to groups based on trivial criteria (e.g., the toss of a coin) after which their behaviors and attitudes toward in- and out-group members are assessed. Although the group allocation is random, its effects can be significantly socially divisive. Minimal group paradigm research with young children has shown that, similar to adults, children tend to overlook positive features of the out-group while at the same time selectively encoding positive information about the in-group (Schug et al., 2013). When given the option, children punish selfish acts of out-group members more than those of in-group members (Jordan et al., 2014), allocate more resources to the in-group and attribute more positive characteristics to the in-group (Dunham et al., 2011). Further, children trust and learn information provided by in-group members more, even if the in-group informant has proven to be unreliable and the out-group informant is reliable (MacDonald et al., 2013). Group-related biases in children as incurred by minimal group allocations are observed in assessments with both explicit measures (e.g., directly asking children how they feel about the out-group) and implicit measures (e.g., matching positive/negative adjectives with in-group/out-group; Dunham et al., 2011). Overall, these findings suggest that groupishness can have a pervasive and profound impact on children's social behaviors, preferences, categorizations, and interactions.

Nevertheless, as the research on the relevance of status in children's group preferences has shown, in-group or similarity-based favoritism may be informed or even overturned under certain conditions. Compared to the large amount of research on how biases are established, there is relatively little research on factors that are associated with the attenuation of children's in-group favoritism. A comparison of 5-, 7-, and 9-year-olds showed that the youngest group displayed more out-group derogation than the older groups, with no difference between 7- and 9-year-olds (Nesdale et al., 2004). This suggests that group-related biases may start to stabilize around 7 years of age. Another study showed that, although 4- to 5-year-old children showed in-group favoritism at baseline, viewing an in-group member behave anti-socially led children to allocate fewer resources and express less liking toward the in-group member (Hetherington et al., 2014). Hence, group stereotyping that young children display under minimal group conditions is not uncompromising; rather, it is strategically attentive to relevant sources of Supplementary Information.

Research in the context of educational and social programs among risk groups and minorities is insightful also. Intergroup contact, for example, can diminish negative attitudes toward racially and ethnically diverse groups in early and middle childhood (e.g., McGlothlin and Killen, 2006; Crystal et al., 2008; Feddes et al., 2009). The effectiveness of intergroup contact appears to stem from the two groups having equal statuses, sharing experiences and forming an overarching group identity that encompasses both groups (Allport, 1954; Rutland and Killen, 2015). Yet, how can these conditions be effectively established? How are such effects achieved? Here we investigate the effect of interpersonal movement synchrony on social bonding across group divides. Evidence from cultural intervention programs supports the view that collective movement, such as in dance, exercise and sport, can reduce intergroup biases and increase bonding and cooperation across groups. For example, school-based community development programs involving dancing in time to rhythms and playing instruments have been shown to increase a range of positive outcomes, including sense of collective identity, understanding of others' cultural values, inclusion of out-group members and feelings of belongingness (Dillon, 2006; Marsh, 2012; for similar results on sports-based programs, see Bailey, 2005). Similarly, training programs conducted with children aged 8–11, which had an emphasis on music production within groups, facilitated emotional empathy (Rabinowitch et al., 2012), sympathy and prosocial attitudes (Schellenberg et al., 2015).

Recent experimental research also has identified positive effects of movement synchrony on bonding and cooperation. In adults, performing synchronous movements, such as walking or tapping in time to the same rhythm as another person, has been shown to enhance feelings of similarity, groupishness, cooperation (Wiltermuth and Heath, 2009; Valdesolo and Desteno, 2011; Reddish et al., 2013) and trust (Launay et al., 2013) among participants. Interestingly, one study found that participants spontaneously synchronized their movements more with minimally constructed out-group members than they did with in-group members (Miles et al., 2011), suggesting that, under certain conditions, movement synchrony may be unconsciously used by individuals to reduce intergroup differences and decrease social distance.

There is mounting evidence also that movement synchrony is linked to pro-sociality and social bonding in children. Infants prefer synchronously-moving social partners to non-synchronously moving partners (Tunçgenç et al., 2015) and help others more if the other person has moved in synchrony with them (Cirelli et al., 2014a). Notably, they do not help a neutral observer who has not performed any movements, suggesting that the pro-social effects of movement synchrony are targeted specifically toward the interactants (Cirelli et al., 2014b), though further research is required to investigate subsequent generalized prosociality toward non-participants (see Carpenter et al., 2013; Reddish et al., 2014). Four-to-six year olds help a game partner more after performing synchronous activities together (Tunçgenç, 2015) and 8–9 year old children report stronger feelings of similarity and closeness after performing rhythmical, synchronous tapping movements with a peer than after comparable non-synchronous movements (Rabinowitch and Knafo-Noam, 2015). Together, these findings affirm the positive social effects of movement synchrony in children. Yet, our understanding of the strength, conditions, and duration of the synchrony effect is still limited. For example, to date, most of the research has investigated children's interactions either with strangers or with one other peer. There are no controlled experimental studies on how movement synchrony operates in more naturalistic group settings among children, the default context of their real-life peer interactions and of the aforementioned intervention and training programs.

Here we aimed to investigate specifically the effect of movement synchrony in facilitating social bonds in an inter-group setting. We hypothesize that movement synchrony between groups facilitates out-group bonding, thereby reducing group-bias between the in-group and the out-group. In order to measure social bonding within and between groups, we used established questionnaires (adapted from Aron et al., 1992; Glass and Benshoff, 2002; Wiltermuth and Heath, 2009; Martin et al., 2012) as well as a new game measure. The game, which we call the Island Game, was an implicit behavioral measure in which physical closeness was used as an indirect measure of social closeness. Physical proximity and approach behavior have long been used as indicators of social closeness and bonding in comparative animal research (e.g., Clay and de Waal, 2013). Social psychology studies conducted with adults have reported strong positive associations between physical proximity and social closeness (IJzerman and Semin, 2010; Fay and Maner, 2012). Inspired by these approaches, the Island Game was developed to measure the effects of synchronous group movement as compared with non-synchronous group movement on group-based preferences by assessing the willingness of children from different groups to be in close proximity with one another (more details below).

## Methods

### Participants

One hundred and two participants (53 female, *M*_*age*_ = 105.25 months, range: 84.10-139.34; *M*_*sync*_ = 104.78, *M*_*non-sync*_ = 105.55) took part in the study. The choice of this age range took account of children's developing motor capabilities. Previous studies have shown that it is only after 7–8 years of age that children can move in synchrony with a rhythm and with other individuals at levels comparable to those of adults (Drake et al., 2000; McAuley et al., 2006). The participants were recruited from local primary schools in [name of place masked for blind review] and came from middle-class, mixed ethnic backgrounds. All of the children were proficient in the language used during testing [language masked for blind review], although 6 children (3 boys) needed the experimenter's (E) help in completing the questionnaires due to reading difficulties. The study received ethics approval from the University's ethics board and, in line with the Declaration of Helsinki, written permission was obtained from the teachers and the parents of the participants prior to testing.

There were six participants in each session (either 4 boys and 2 girls or 2 boys and 4 girls), split into two arbitrary groups (either 2 boys and 1 girl or 1 boy and 2 girls in both groups). Groups therefore resembled real-life mixed-sex groups, without sex being introduced as another grouping factor as neither group or session consisted exclusively of girls or boys. The within-session age difference among children ranged between 6 months 4 days and 12 months 16 days, a statistically insignificant difference, *F*_(1, 15)_ = 0.003, *p* = 0.96. More detailed descriptive statistics on the number of participants by condition are provided in Table 1.

**Table 1:** **Descriptive statistics of the sample by condition and in total**.

		**Synchrony**	**Non-synchrony**	**Total**
Sex (*n* = number of participants)	Female	27	26	53
	Male	21	28	49
Tempo (*n* = number of sessions)	585ms	8	9	17
	555ms	8	9	17
Movement Set (*n* = number of sessions)	Set 1	8	9	17
	Set 2	8	9	17

### Materials and procedure

#### General set-up and procedure

**Figure 1:**
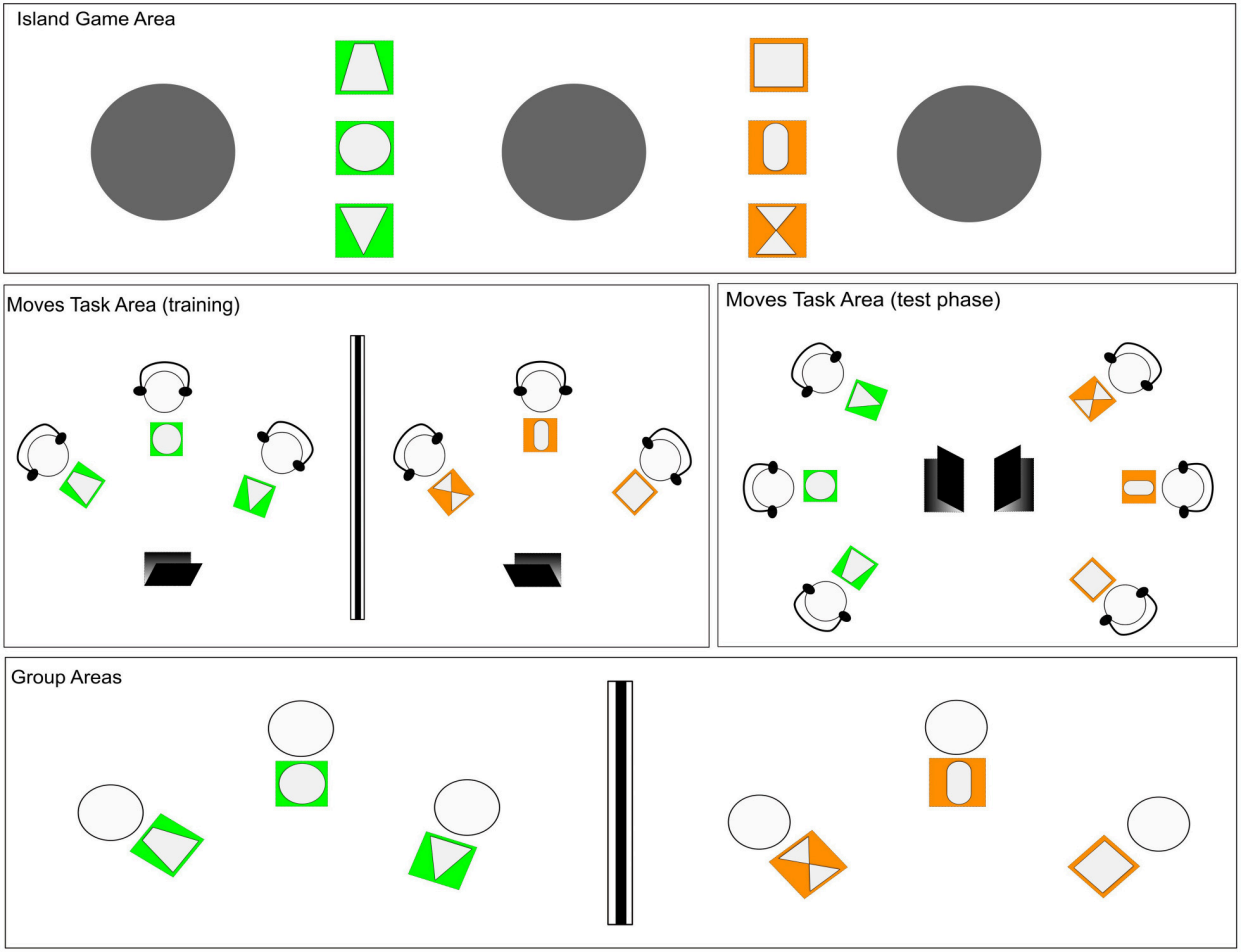
**Overview of the study set-up**. Phase1, Phase 2, and Phase 5 took place in the Group Areas, Phase 3 in the Moves Task Area and Phase 4 in the Island Game Area.

Each session consisted of 5 phases: (1) minimal group formation, (2) pre-test questionnaires, (3) Moves Task, (4) Island Game, and (5) post-test (long) questionnaire. Three separate areas were created in the experiment room to accommodate the different tasks (see Figure 1).

Upon arrival, children were first assigned to one of two groups: the orange group or the green group. Once the groups were formed, children wore vests that matched their group's color and were directed to their group's area. In the Group Areas, there were 3 mats (of matching color to the group's color) with individual shapes printed on them. The shapes were used to identify the children individually. Cards with these shapes printed on them were used throughout the experiment to indicate each child's spot in a task or game. The Group Areas were used for a warm-up activity and for children to complete the questionnaires. The Moves Task took place in an area adjacent to the Group Areas. A tall room divider that occluded visual access separated the Moves Task Areas of the two groups during the training phase. The children learned and practiced the Moves Task within their groups with the room divider present. For the test phase, E removed the divider and the children performed the Moves Task while facing the other group. Following the Moves Task phase, the children were taken to the Island Game Area, where they played the Island Game. Finally, children returned to their Group Areas to complete post-test questionnaires.

#### Phase 1: Minimal group formation

To determine the group composition, children drew cards from a ballot bag. Although the drawing of cards seemed random, in reality, the cards were assorted in such a way to ensure the correct male-female ratio in each group, i.e., by fixing the order of drawing the cards. Cards were colored either orange or green and had certain shapes on them, which were used to identify the children individually (colors and shapes counterbalanced across conditions). To further establish identification with their group, children wore color-coded group vests. They then took their seats on their group's mats and did a warm-up activity. The warm-up activity required the children to work together with their group to draw a group flag that was then hung on the board in their Group Area.

#### Phase 2: Pre-test questionnaires

At their individual positions in their Group Areas, children were asked to complete two brief questionnaires to assess how bonded they were to their in-group and to the out-group; the In-group Bonding (IB_pre-*test*_) and Out-Group Bonding (OB_pre-*test*_) questionnaires, respectively. For a list of the questionnaire items, see Table S1. Both IB_pre-*test*_ and OB_pre-*test*_ consisted of three 5-point Likert type questions each (where 1 = Strongly disagree, 2 = Disagree, 3 = Don't agree or disagree, 4 = Agree, 5 = Strongly agree). The questions were adapted from questionnaires previously used to examine sports teams' bonding in children (Glass and Benshoff, 2002; Martin et al., 2012). Brief instruction on how to use the Likert scale was given at the beginning. Children completed the questionnaires individually and privately in their own time.

#### Phase 3: Moves task

On completion of the pre-test questionnaires, children were brought to the Moves Task Area. This task required the children to perform whole-body movements in time to beats that they heard from their headphones.

Training videos, created by the first author (B.T.), were used. In these, she instructed children on how to perform the moves in time to the beat and demonstrated the moves. All of the moves were basic leg and arm movements such as stepping to the side and stretching or swinging the arms. None required any prior experience and they were selected so as to avoid evoking a particular meaning (e.g., such as in clapping, marching, etc.). For a sample of the moves video used, please refer to the Supplementary Materials video.

For the beats, two auditory tracks of drumbeats (585 and 555 ms) were created using Garageband software. Using Silent Disco technology, each computer was connected to a separate audio channel, from which the drumbeats were presented to children's individual wireless headphones. This way, the two groups in each session could receive their own visual (instruction videos for the moves) and auditory stimuli (the beats presented through the two audio channels) simultaneously without knowing what stimulus the other group was receiving. In the synchrony condition, both groups were presented with the same movement set and the same beats, while in the non-synchrony condition different movement sets and different tempi beats were presented to the two groups. To increase the contrast across conditions, two different movement sets were used, each comprising three moves.

Videos were shown to the children via 13″ computer screens, a separate one for each group. The training videos lasted for just under 6 min. In the videos, the instructor first demonstrated the moves in a step-wise fashion one by one. Following each demonstration, she asked the children to join her in doing the move in time to the beats. When individual training for all three moves was completed, the moves were practiced one final time for the whole duration of the auditory track that was later presented in the test phase. E observed the children during training; all of the children could proficiently perform the moves following the beats by the end of the training.

After the training was over, E instructed the children that they would do the moves once more, this time facing the other group. She removed the room divider between the groups and positioned the children in a crescent shape around the computer screens, so that all children could see all the other group members, their own group members and their video at the same time (see Figure 1). In order to eliminate potential memory demands, an instruction-free video of E performing the moves was provided during the test phase too. The test phase lasted for approximately 3 min.

#### Phase 4: Island game

The Island Game took place immediately after the Moves Task test. In this game, three islands (charcoal-colored mats of 100 cm diameter) were placed on the floor, each spaced approximately 2 m from the adjacent island. One group of children (orange or green) was positioned in the space between two of the islands and the other group of children was positioned in the space between the other two islands. Two of the islands were therefore closer to either one of the groups and one island in the middle was equally close to both groups (see Figure 1). Children's individual positions were determined via the shape cards on the floor.

The game required that the children would start from a crouching position and would quickly run to an island of their choice on the experimenter's signal. At the start of each trial, the children were told to crouch on their dedicated spots with eyes closed and their face to the floor. They maintained this position until E counted down from 3, at which point E announced, “Go!” and the children jumped up and ran to the island that they chose. Choosing to go to an island that was closer to one's own group vs. the other group was taken as a reflection of social closeness to one's own group vs. the other group. The Island Game was repeated 6 times and children's starting positions within their group “zone” were shuffled each time. After the Island Game was finished, children went back to their Group Area and completed the post-test questionnaires.

#### Phase 5: Post-test (long) questionnaire

The post-test questionnaire comprised of three parts. Unless otherwise stated, all questions were answered on a 5-point Likert type scale (1 = Strongly disagree, 2 = Disagree, 3 = Don't agree or disagree, 4 = Agree, 5 = Strongly agree). A list of the questionnaire items is provided on Table S1.

The first set of questions concerned children's experiences of the activities that they had just done (perceived difficulty, success and enjoyment). The second set of questions was similar to the pre-test questionnaires; items assessed how bonded children felt toward their in-group and the out-group. We named these questionnaires IB_long_ and OB_long_. Within both IB_long_ and OB_long_, 3 of the items were identical to those in the pre-test questionnaires, IB_pre-test_ and OB_pre-test_. These 3 items (IB_post-test_ and OB_post-test_) were analyzed separately to assess how children's bonding levels changed before and after the Moves Task (more details follow in the Results section). The items in IB_long_ and OB_long_ were adapted from existing measures of sports team bonding and bonding questions used in adult synchrony research (see Table S1 for sources for each item).

The third section of the post-test questionnaires was an adapted version of the pictorial Inclusion of Other in Self (IOS) scale, used to assess participants' perceived relationship to the in-group and to the out-group (Cameron et al., 2006). In this scale, two circles representing the participant and the group were displayed with increasing degrees of overlap (from entirely separated to entirely overlapping), yielding five response options. Circles were annotated with stickman figures representing the child (labeled “YOU”) and the group (labeled “YOUR GROUP” or “OTHER GROUP”). Participants marked one of the five options that best represented how close or how distant they felt toward the group under question. Instructions on how to interpret the IOS scale were provided to all children at the beginning of the post-test questionnaire phase.

The post-test questionnaires ended with one question (Q48), which asked the children whom they would like to choose as playmates if they were to do some more activities later. Children indicated their response by choosing one of the four multiple-choice answers: (a) 2 people from my group, (b) 2 people from the other group, (c) 1 person from my group and 1 person from the other group, and (d) Any 2 people—I don't mind.

When finished, children were thanked and dismissed from the study. The Moves Task and Island Game phases of all sessions were video recorded for coding purposes.

## Coding and data preparation

### Moves task

A coder blind to the hypotheses and conditions watched all of the Moves Task test phases of the videos and rated them for synchrony. First, the coder assessed how synchronously the two groups moved within a session by giving that session a rating from 1 to 7, where 1 = not at all synchronous and 7 = perfectly synchronous, and then made a blind guess as to which condition the video belonged to. Ratings for the synchrony condition (*M*_*sync*_ = 5.75) were significantly higher than ratings for the non-synchrony condition (*M*_*non-sync*_ = 3.22), *t*_(16)_ = −5.26, *p* < 0.0001. Condition guess accuracy was 100%, binomial *p* < 0.0001.

### Bonding questionnaires

Factor analyses were conducted for the six Likert type questionnaires: IB_pre-test_, OB_pre-test_, IB_post-test_, OB_post-test_, IB_long_, and OB_long_. Preliminary tests and correlational analyses confirmed that the questionnaires were suitable for factor analysis (for inter-item correlations see Figures S1–S3; for the other tests see Table S2). From the results of the Principal Component Analysis (PCA), only one component was extracted from each questionnaire, which we interpret as a single, composite construct of social bonding. Only items with a factor loading of >0.4 were retained. Consequently, two items were dropped from the OB_long_ questionnaire, namely the items “I don't like my group” and “I feel bad about my group”. For all other questionnaires, all of the items were retained. The detailed results of the PCAs and internal consistency values of each questionnaire can be found in Tables S3 and S4. Given the high internal consistencies of the questionnaires, responses across items for each questionnaire were averaged for each participant and these mean scores were used in subsequent analyses.

### IOS scales

For the IOS scales, children received a score ranging from 1 to 5 depending on the option chosen, with higher scores indicating higher self-group overlap.

### Island game

Participants' island choices were categorically coded as 0, 1 or 2, where 0 = the island closest to the participant's own group and furthest from the other group (“Own Group Island”), 1 = the island in the middle (equidistant from the group islands and to participants from each group), and 2 = the island closest to the other group and furthest from the participant's own group (“Other Group Island”). Reliability analysis on children's Island Game choices, conducted with two coders who were blind to conditions and one who was blind to the hypotheses, revealed good agreement, *r* = 0.87.

Recognizing the potential for island choices to be influenced by the other participants in this group task, we analyzed the Island Game data for Intraclass Correlations (ICC). When all six trials were included in analysis, average intra-session variance differed from the overall variance, *ICC* = 0.24, CI [0.526, 0.720], *F*_(1, 100)_ = 4.27, *p* = 0.03, suggesting interdependence among children's responses within trials. Therefore, we assessed ICC for the first trial only, in which children made their initial island choices and which we would therefore expect to be uninfluenced by their own and others' previous choices. The interdependence in children's responses disappeared when only the first trial responses were analyzed, *ICC* = 0.009, CI [−0.014, 0.967], *F*_(1, 100)_ = 1.16, *p* = 0.28 (see Supplementary Materials for ICC scores by trial and condition). Hence, to avoid problems of group-level interdependence in subsequent main analyses, only first trial responses were used.

All analyses were performed using R software (R Core Team, 2013) and following the recommendations of Field et al. (2012).

## Results

### Minimal group bias

A manipulation check for the minimal group effect confirmed that, at baseline (i.e., before performing the Moves Task) children overall reported higher bonding toward their in-group (*M*_*IB*_ = 4.37) than toward the out-group (*M*_*OB*_ = 3.22), *F*_(1, 95)_ = 73.41, *p* < 0.0001, η^2^ = 0.44. Being in the synchronous vs. non-synchronous condition did not influence children's pre- Moves Task scores on IB_pre-test_, *M*_*sync*_ = 4.40, *M*_*non-sync*_ = 4.35, *F*_(1, 94)_ = 0.22, *p* = 0.64, or on OB_pre-test_, *M*_*sync*_ = 3.21, *M*_*non-sync*_ = 3.22, *F*_(1, 94)_ = 0.01, *p* = 0.99. Within both synchrony and non-synchrony conditions, boys scored lower on out-group bonding questions than girls, *M*_*boys*_ = 2.89, *M*_*girls*_ = 3.52, *F*_(1, 94)_ = 8.04, *p* = 0.006. No differences in in-group bonding were found between boys and girls. Further, a main effect of age was found on both in-group and out-group questionnaires, in-group: *F*_(1, 94)_ = 4.65, *p* = 0.03; out-group: *F*_(1, 94)_ = 3.66, *p* = 0.06. With increasing age, a gradual decrease was observed in inter-group biases. However, Bonferroni-adjusted pairwise comparisons revealed no significant differences between any of the age groups between 7 and 11, all *p*s >0.05. No effect of group color was found in any of the pre-test bonding questionnaires.

### Synchrony effects

#### Bonding questionnaires

First, data were checked for any differences across conditions in children's experiences with the games. Children in the synchrony and non-synchrony conditions found the Moves Task similarly easy, *M*_*sync*_ = 4.58, *M*_*non-sync*_ = 4.32, *F*_(1, 100)_ = 2.64, *p* = 0.11. Enjoyment ratings were also similarly high across conditions for the Moves Task, *M*_*sync*_ = 4.25, *M*_*non-sync*_ = 4.24, *F*_(1, 100)_ = 0.002, *p* = 0.96, and the Island Game, *M*_*sync*_ = 4.46, *M*_*non-sync*_ = 4.37, *F*_(1, 100)_ = 0.24, *p* = 0.62. Neither short or long post-test questionnaires were significantly influenced by children's age, sex, group color, tempo, and movement set, all *p*s > 0.05.

**Figure 2:**
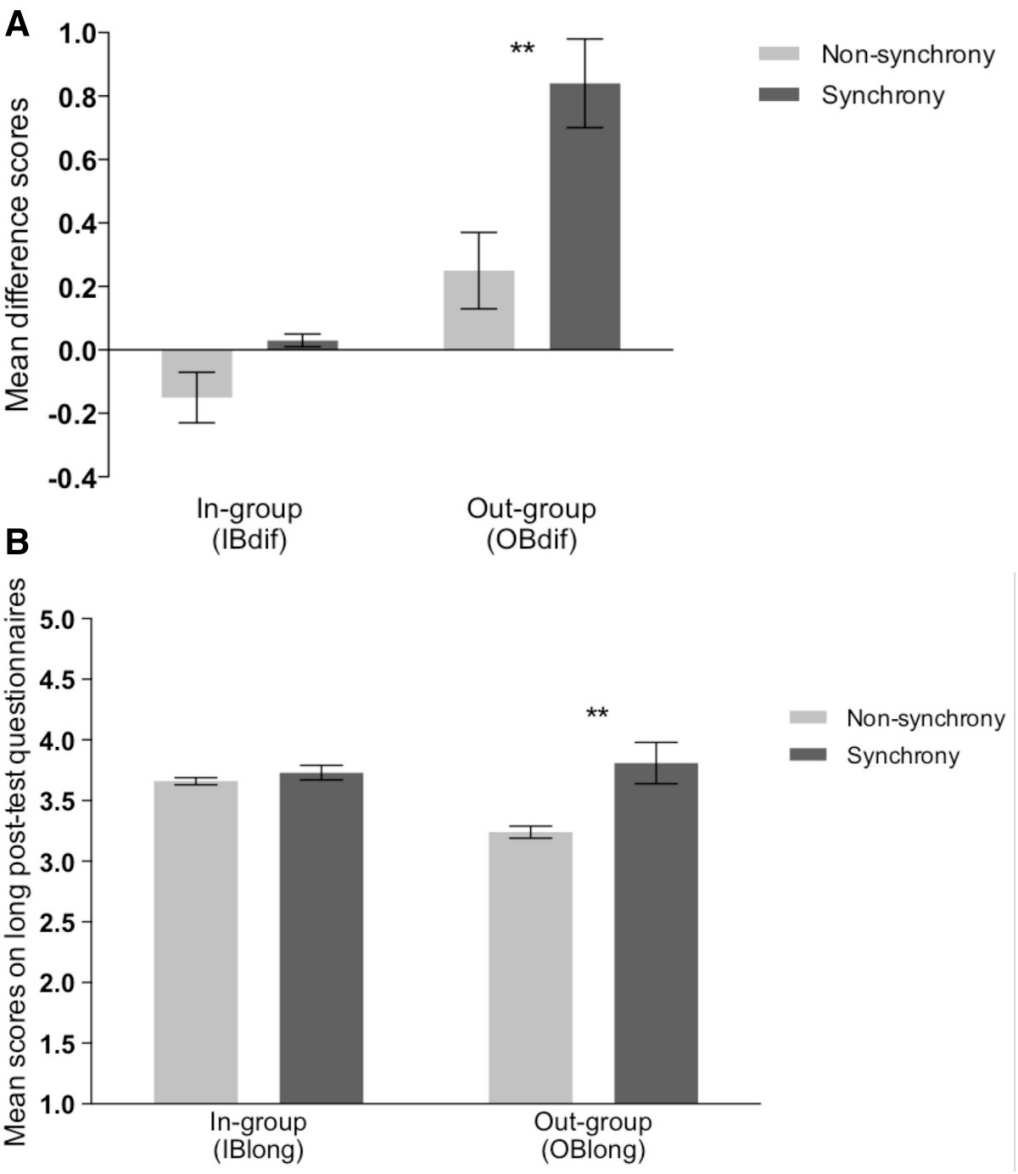
**Mean scores on bonding questionnaires by condition**. **(A)** Mean difference between pre-test and post-test questionnaire responses by condition. **(B)** Means of the long post-test questionnaire responses, IB_long_ and OB_long_, by condition. ^**^p < 0.05.

Next, we examined the effect of movement synchrony on children's in-group and out-group bonding in the short pre-test and post-test questionnaires and the long post-test questionnaires. For the short questionnaires, difference scores were calculated by subtracting participants' post-Moves Task scores from their pre-Moves Task scores on the short IB and OB questionnaires, i.e., IB_dif_ = IB_post-test_ − IB_pre-test_, by which higher positive scores indicate greater increase in bonding. The exact means of children's IB and OB scores before and after the Moves Task are provided in Table 2. No effect of condition was found on the change in children's bonding with the in-group (IB_*dif*_), *M*_*sync*_ = 0.03, *M*_*non-sync*_ = −0.15, *F*_(1, 94)_ = 2.02, *p* = 0.16. However, as predicted, OB_dif_ was higher for children in the synchrony condition (*M*_*sync*_ = 0.84) than for children in the non-synchrony condition (*M*_*non-sync*_ = 0.25), *F*_(1, 94)_ = 9.16, *p* = 0.003, η^2^ = 0.09 (see Figure 2A). Separate paired-samples *t*-tests within each condition confirmed that the increase in out-group bonding was significant for the synchrony condition, *t*_(41)_ = −5.47, *p* < 0.0001, *d* = 0.84, but not for the non-synchrony condition, *t*_(53)_ = −1.58, *p* = 0.12.

**Table 2:** **Means of the short versions of the pre-test and post-test questionnaires, IB and OB, by condition**.

	**In-group (IB)**		**Out-group (OB)**	
	**Pre-test**	**Post-test**	**Pre-test**	**Post-test**
Synchrony	4.40	4.43	3.21	4.04
Non-synchrony	4.35	4.20	3.22	3.46

Similar results were obtained for the long post-test questionnaires: paired *t*-tests revealed that there was no significant effect of condition on in-group bonding (IB_long_), *M*_*sync*_ = 3.73, *M*_*non-sync*_ = 3.66, *F*_(1, 100)_ = 0.36, *p* = 0.55, but bonding to the out-group (OB_long_) was significantly higher in the synchronous than the non-synchronous condition (see Figure 2B), *M*_*sync*_ = 3.81, *M*_*non-sync*_ = 3.24, *F*_(1, 100)_ = 6.76, *p* = 0.01, η^2^ = 0.06. Further, following synchronous movement, levels of bonding to the in-group (IB_long_) and out-group (OB_long_) were indistinguishable, M_IB_ = 3.73, M_OB_ = 3.81, *t*_(47)_ = −0.71, *p* = 0.48. In the non-synchrony condition, the difference between in-group bonding (IB_long_) and out-group bonding (OB_long_) trended in the predicted direction, M_IB_ = 3.66, M_OB_ = 3.34, *t*_(53)_ = 1.72, *p* = 0.09, *d* = 0.23.

These results support our hypothesis that, compared to non-synchronous movement, performing synchronous movements increases out-group bonding among participants. Results are further corroborated by condition-wise differences in the reported success of the in-group and out-group Moves Task performances. Ratings for how successful children thought their own group was in performing the Moves Task did not differ significantly between the conditions, *M*_*sync*_ = 4.38, *M*_*non-sync*_ = 4.28, *F*_(1, 100)_ = 0.27, *p* = 0.60. However, children in the synchrony condition rated the other group as more successful in the Moves Task (*M*_*sync*_ = 4.15) than did children in the non-synchrony condition (*M*_*non-sync*_ = 3.56), *F*_(1, 100)_ = 6.36, *p* = 0.01, η^2^ = 0.06.

#### IOS scales

**Figure 3:**
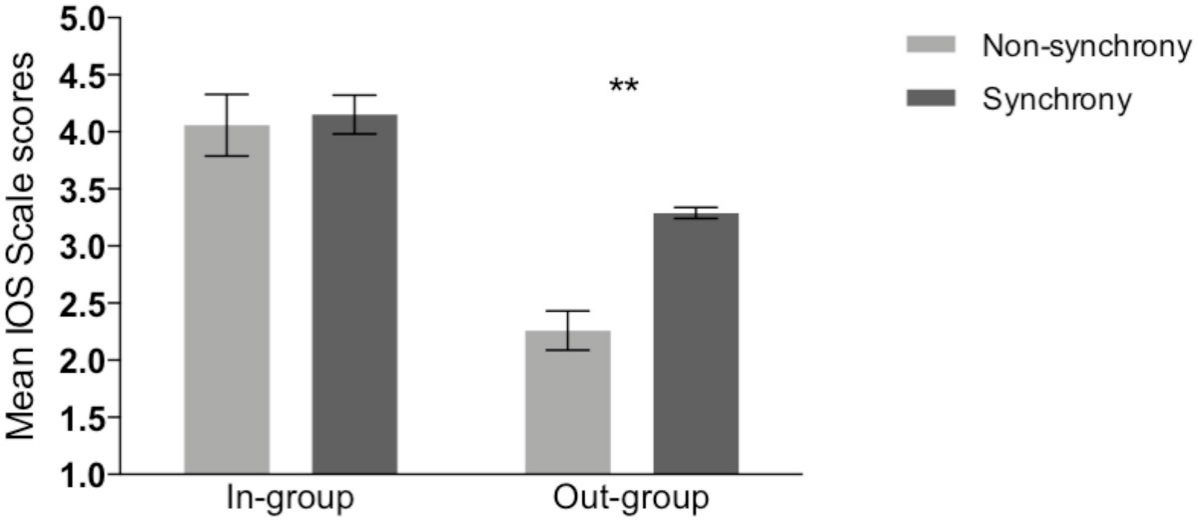
**Mean scores on in-group IOS and out-group IOS scales by condition**. ^**^p < 0.05.

IOS responses were analyzed using ordinal logistic regression to test the prediction that scores for the out-group would be higher in the synchrony condition than in the non-synchrony condition. Data were checked for the proportional odds assumption; no violation was found for either the in-group IOS, *X*${}_{\left(3\right)}^{2}$ = 5.95, *p* = 0.11, or the out-group IOS scale, *X*${}_{\left(3\right)}^{2}$ = 3.35, *p* = 0.34. Hence, ordinal logistic regression analyses with condition, age, sex, group color, tempo, and movement set as predictors were conducted. None of the predictors were found to have an effect on children's responses for the in-group IOS scale, all *p*s > 0.05. This indicates that condition did not influence how close children felt toward their in-group following the Moves Task, which is in line with the previous questionnaire findings. For the out-group IOS scale, only condition significantly predicted responses; the odds of children in the synchrony condition scoring higher on the out-group IOS scale was 3.98 times that of children in the non-synchrony condition, *X*${}_{\left(1\right)}^{2}$ = 13.52, *p* < 0.0001, 97.5% CI, 1.93–8.43. Moreover, within both the synchrony and the non-synchrony conditions, the differences between in-group and out-group IOS scores were significant, suggesting that even after synchronous movement performance, some in-group favoritism remained (see Figure 3), synchrony condition: *t*_(47)_ = 3.90, *p* < 0.001, *d* = 0.56; non-synchrony condition: *t*_(53)_ = 7.83, *p* < 0.0001, *d* = 1.07.

Finally, children's responses to the final item on the post-test questionnaire (Q48) were assessed. This item asked whether the children would choose in-group or out-group members as playmates for a hypothetical future encounter. No difference was found across conditions, *X*${}_{\left(3\right)}^{2}$ = 5.02, *p* = 0.17. Table 3 shows the descriptive statistics of children's responses to Q48.

**Table 3:** **Frequencies (n) of children's responses to Question 48 (“If we came back to do more activities another day, who would you choose to do them with?”) by condition**.

**Response options for Q48**	**Synchrony**	**Non-synchrony**	**Total**
2 people from my group	9 (19%)	9 (17%)	18 (18%)
1 person from my group and 1 person from the other group	10 (21%)	6 (11%)	16 (16%)
2 people from the other group	11 (23%)	23 (43%)	34 (33%)
Any 2 people - I don't mind	18 (37%)	16 (29%)	34 (33%)

*Column percentages are provided in parentheses next to the frequencies*.

#### Island game

Island Game analyses assessed whether children in the synchrony condition were more likely than children in the non-synchrony condition to choose the “Other Group Island”. Table 4 shows the descriptive statistics of children's Island Game responses by condition. The data did not meet the assumptions of multinomial logistic regression (specifically, the high leverage assumption of multinomial logistic regression, i.e., zero cases in the Non-synchronous/ the “Other Group Island” cell, as can be seen in Table 4). Hence, a chi-square test was run to analyze the effects of condition (synchronous vs. non-synchronous) on children's island choices. The result showed a significant effect of condition with a large effect size, *X*${}_{\left(2\right)}^{2}$ = 13.29, *p* = 0.001, φ = 0.36. Children in the non-synchrony condition were 1.69 times more likely to go to the “Own Group Island” than children in the synchrony condition. Moreover, while no child in the non-synchrony condition chose to go to the “Other Group Island”, 19% of the children in the synchronous condition chose to do so. In both conditions, children were roughly equally likely to choose the middle island. No significant associations were found between children's age, sex, group color, tempo, and movement set and island choices, all *p*s > 0.05 (see Table S5). These findings align with the questionnaire results above; children's behavioral responses in the Island Game strongly predicted their scores on the out-group IOS Scale, *F*_(1, 100)_ = 12.00, *p* = 0.0008, *R*^2^ = 0.11, and out-group bonding questionnaire (OB_long_): *F*_(1, 100)_ = 14.51, *p* = 0.0002, *R*^2^ = 0.13, giving confidence in the Island Game as a novel behavioral measure of social bonding and preference in children.

**Table 4:** **Frequencies (n) of children's Island Game responses by condition**.

	**Synchrony**	**Non-synchrony**	**Total**
“Own Group Island” (the island closest to own group/furthest from other group)	17 (35%)	32 (59%)	49 (48%)
Middle island (the island in the middle, equidistant to both groups and the other islands)	22 (46%)	22 (41%)	44 (43%)
“Other Group Island” (the island closest to the other group/furthest from own group)	9 (19%)	0 (0%)	9 (9%)

*Column percentages are provided in parentheses next to the frequencies*.

## Discussion

This experiment investigated whether synchronous movement reduces inter-group biases and increases bonding among groups. In line with previous research on minimal group effects, we found that children initially displayed in-group favoritism following group allocation and a brief identity-building activity. Subsequent performance of movements in synchrony, as compared to non-synchronous movement, with the opposing group significantly increased out-group bonding. After synchronous movement, there was no difference between bonding to the in-group and the out-group, as measured by the long questionnaire items, though pictorial IOS responses suggest some lingering in-group bias in perceived closeness. Importantly, while synchrony appears to have increased out-group bonding, non-synchrony did not significantly affect bonding toward the out-group. This suggests that merely getting together and moving in the same space has little effect on bonding between groups; rather, it is synchronous movement specifically that brings about a significant positive change.

In addition to questionnaire measures, we also used a novel behavioral measure, the Island Game, as a gauge of social bonding. Analyses from both the questionnaire and behavioral measures revealed greater out-group bonding in the synchrony condition compared to the non-synchrony condition. Since the post-test questionnaires clearly show that in-group bonding was as high after the Moves Task as it was at baseline, the condition-wise effects on Island Game behavior more likely reflect increased out-group bonding, rather than reduced in-group bonding. The Island Game took physical closeness as a proxy for social closeness. This idea finds its roots in established measures of affiliation in primate communities (Clay and de Waal, 2013) and in social psychology studies revealing a relation between physical and social closeness (IJzerman and Semin, 2010; Fay and Maner, 2012). The design was based on playground games that children commonly enjoy and therefore offers high ecological validity (Torbert and Schneider, 1993; Pica, 2011). Children's ratings indicate that the game was fun to play. Importantly, behavioral responses in the Island Game strongly predicted self-report measures. Unlike the questionnaire measures, game instructions do not entail or draw attention to group identity or group competition. The Island Game therefore potentially captures implicit aspects of inter-group biases and social closeness. One limitation of the game, however, is the potential for interdependence; after the first trial, children's responses in the following iterations of the game may have been prone to be influenced by other children's choices. Methodological solutions to reduce such influences could be considered in future developments of the design. Alternatively, larger sample sizes could allow multilevel analysis. These observations notwithstanding, convergent findings with the questionnaire responses confirm that the Island Game successfully measures group bonding with a single-trial assessment.

Several psychological mechanisms may play a role in creating the observed positive effects of movement synchrony on group social bonding. Previous research has shown that people who view their partners more positively tend to spontaneously synchronize their movements with them more than do partners with weaker rapport (Miles et al., 2010). Moving in synchrony can also lead to increased perceptions of similarity (Wiltermuth and Heath, 2009; Valdesolo and Desteno, 2011; Rabinowitch and Knafo-Noam, 2015). Thus, in the current study, performing the same movements in time to the same beats with the out-group might have enhanced rapport and feelings of similarity, thereby alleviating relative negative bias against the out-group. Synchronous movement also increases perceived entitativity (i.e., having the properties of a single, united entity; Campbell, 1958). People who move synchronously with each other are perceived as having higher entitativity (Lakens, 2010) and perceptions of entitativity are mediated by psychological attributions, such as assuming that synchronous partners feel the same, or that they like each other more (Lakens and Stel, 2011). Similarly, from a first-person perspective, interactants report feeling more connected and as part of the same team upon performing synchronous movements (Wiltermuth and Heath, 2009; Wiltermuth S., 2012; Wiltermuth S. S., 2012; Cohen et al., 2013). The results of the present study support the entitativity account, as evidenced by reports of increased connectedness, togetherness and closeness in the synchrony condition. Thus, movement synchrony potentially forges social bonds and fosters more positive attitudes among synchronizing partners.

From a broader evolutionary perspective, it has been suggested that movement synchrony may have facilitated cooperation in large human societies, where bonding with genetically unrelated individuals presented unique challenges (Dunbar and Shultz, 2010). By moving in time to a shared rhythm, personal identities are thought to merge into a unified group identity (McNeill, 1995), accompanied by feelings of collective joy (Ehrenreich, 2006) and collective effervescence (Durkheim, 1915/1965). There is a growing amount of empirical support for the facilitatory effects of movement synchrony on emotion sharing and empathy (Cross et al., 2012; Valdesolo and Desteno, 2011). Understanding and sharing the emotional states of others promotes pro-social behaviors both in human children (Dunfield, 2014) and in non-human primates (Melis et al., 2006; Hare et al., 2007). Therefore, movement synchrony may be a fundamental mechanism in social bonding, serving to mitigate emotional tension among individuals and groups and bond them together within a collective identity.

The current study contributes to our understanding of the developmental origins of both intergroup bias and the social bonding effects of synchronous movement. In revealing an effect of synchrony on out-group bonding, the findings shed light on the flexibility of inter-group biases in middle childhood years, an underexplored question in the social developmental literature (though see: Hetherington et al., 2014). Notably, at baseline, boys and younger children held a more negative attitude toward the out-group than girls and older children (for similar results, see Buttelmann and Böhm, 2014). Measures taken after the Moves Task reveal that synchrony also erased these differences. That synchronous movement, but not non-synchronous movement, can have these effects could usefully inform social intervention programs, especially in cases where cohesion across groups is challenged by prior social, cultural or economic divides. In this study, minimal group construction produced differences in reported in- vs. out-group bonding; yet, it should be noted that out-group bonding was still in the positive range of the scale (for comparable results on minimal group studies with children, see: Nesdale et al., 2004; Dunham et al., 2011). Relatedly, the increase in out-group bonding, as assessed by the questionnaires, was modest and statistical analyses revealed small effect sizes. Therefore, it remains to be seen whether the positive social effects of movement synchrony could mitigate negative sentiment or even hostility toward real-world out-groups where social divides are strongly entrenched.

Different types of synchronous movements included in bonding activities could yield variable effects also. Here, we manipulated both the content of the moves and the timing of the beats to attain maximal difference across conditions. Perhaps changing the timing alone could facilitate out-group bonding, as is shown to be the case in children's dyadic interactions (Rabinowitch and Knafo-Noam, 2015; Tunçgenç, 2015). Moreover, the movements in the present study were contextualized within a joint physical play context. Previous research has indicated the social benefits of physical activity and exercise in children's play (Biddle and Asare, 2011; Barkley et al., 2014). In the future, it will be important to explore how the bonding effects of synchrony manifest in other cross-group contexts, such as musical interactions (e.g., Kirschner and Tomasello, 2010), sport, exercise, and joint physically active play. Finally, it would be productive to investigate how movement synchrony influences bonds across group members in inter-group settings in adults. Despite the vast literature on dyadic social bonding effects of synchrony in adults, there have been relatively fewer studies on synchrony-induced bonding within groups (e.g., Cohen et al., 2013; Reddish et al., 2013; Davis et al., 2015; Tarr et al., 2015). To our knowledge, no study has examined how synchrony influences inter-group dynamics or group-related biases.

From infancy to adulthood, group-based differences strongly influence social preferences, attitudes, and behaviors toward others. In this study, we showed that a brief, fun movement game, when performed in synchrony with an opposing group, led to increased bonding across group divides. The findings advance our understanding of the links between motor, cognitive and social development in middle childhood years. The new behavioral measure developed, the Island Game, as well as the questionnaires used, offer valid, reliable measures of social bonding that are convenient to administer to young children. Results can helpfully inform social and educational interventions that aim to increase social closeness and cooperation in group settings. Future research should continue to investigate the mechanisms by which movement synchrony forges social bonds, the range of contexts and activities in which these effects work, and the relevance of synchrony for interventions across real-world divides.

## Author contributions

BT and EC designed the study, analyzed the data and wrote the paper; BT collected the data.

## Funding

This research was supported by a British Academy Fellowship to EC (No. MD130076).

### Conflict of interest statement

The authors declare that the research was conducted in the absence of any commercial or financial relationships that could be construed as a potential conflict of interest.
