# Corrigendum: Movement Synchrony Forges Social Bonds across Group Divides

**DOI:** 10.3389/fpsyg.2016.01737

**Published:** 2016-11-04

**Authors:** Bahar Tunçgenç, Emma Cohen

**Affiliations:** School of Anthropology and Museum Ethnography, Institute of Cognitive and Evolutionary Anthropology, University of OxfordOxford, UK

**Keywords:** children, in-group attitudes, minimal group paradigm, out-group attitudes, prosociality, social bonding, cooperation, movement synchrony

Reason for corrigendum:

In the original version of our article, there was a typographical error in the Participants section. The name of place and university where the study was conducted and the language of the study were masked for blind review and remained masked in the published version. The relevant sentences should read as (changes marked in bold): “The participants were recruited from local primary schools in **Oxfordshire, UK** and came from middle-class, mixed ethnic backgrounds. All of the children were proficient in **English**, although 6 children (3 boys) needed the experimenter's (E) help in completing the questionnaires due to reading difficulties. The study received ethics approval from the **University of Oxford**'s ethics board and, in line with the Declaration of Helsinki, written permission was obtained from the teachers and the parents of the participants prior to testing.”

## Author contributions

BT and EC designed the study, analyzed the data, and wrote the paper; BT collected the data.

## Funding

This research was supported by a British Academy Fellowship to EC (No. MD130076).

### Conflict of interest statement

The authors declare that the research was conducted in the absence of any commercial or financial relationships that could be construed as a potential conflict of interest.

