# Supplementary material for: Movement Synchrony Forges Social Bonds across Group Divides
Source: Front Psychol. 2016 May 27;7:782. doi: 10.3389/fpsyg.2016.00782 (PMC4882973; doi:10.3389/fpsyg.2016.00782)
Supplement: Supplementary file 1 [file Table1.DOCX]

| Table S1. *Questionnaire items as they appeared in children’s question booklets.* *Questions 1-4 and 11-14 were used for identification purposes. Questions 33 and 47 were IOS Scales, answered by choosing one of the five pictorial options. Question 48 was answered using one of the four options provided in the question itself. The rest of the items were answered by circling around one of the options on a 5-point Likert type scale, where the labels were indicated as “Strongly disagree, Disagree, Don’t agree or disagree, Agree, Strongly agree”. Asterisks indicate the resource from which the item was adapted from: ^*^Wiltermuth & Heath, 2009; ^**^Martin, Carron, Eys & Loughead, 2012; ^***^ Glass & Benshoff, 2002.* | |
| --- | --- |
| **Pre-test Questionnaires** | |
| 1. Your Name | |
| 2. Your Shape | |
| 3. Your Group’s Colour | |
| 4. The Other Group’s Colour | |
| 5. I like my group^***^ | 8. I like the other group^***^ |
| 6. I would enjoy helping my group^**^ | 9. I would enjoy helping the other group^**^ |
| 7. My group is cool^**^ | 10. The other group is cool^**^ |
| **Post-test Questionnaires** | |
| 11. Your Name | |
| 12. Your Shape | |
| 13. Your Group’s Colour | |
| 14. The Other Group’s Colour | |
| 15. Performing the moves was easy for me. | |
| 16. I enjoyed doing the moves. | |
| 17. I think my group was successful in doing the moves. | |
| 18. I think the other group was successful in doing the moves. | |
| 19. I enjoyed playing the Island Game. | |
| 20. I feel connected to my group^*^ | 34. I feel connected to the other group^*^ |
| 21. I feel on the same team with my group^*^ | 35. I feel on the same team with the other group^*^ |
| 22. I feel good about my group^**^ | 36. I feel good about the other group^**^ |
| 23. I think I am similar to my group in general^*^ | 37. I think I am similar to the other group in general^*^ |
| 24. I share the same goals as my group^***^ | 38. I share the same goals as the other group^***^ |
| 25. I feel bad about my group^**^ | 39. I feel bad about the other group^**^ |
| 26. I work well together with my group ^***^ | 40. I work well together with the other group^***^ |
| 27. I am united with my group^***^ | 41. I am united with the other group^***^ |
| 28. I don't like my group^***^ | 42. I don't like the other group^***^ |
| 29. I would stick together with my group during challenges^**^ | 43. I would stick together with the other group during challenges^**^ |
| 30. I like my group^***^ | 44. I like the other group^***^ |
| 31. I would enjoy helping my group^**^ | 45. I would enjoy helping the other group^**^ |
| 32. My group is cool^**^ | 46. The other group is cool^**^ |
| 33. Think about yourself and your feelings for your OWN GROUP (the one that shares your colour). How close or distant do you feel to your own group? Choose from below ONE picture (A - E) that best represents how close or distant you feel to your own group. Put a cross in the ☐ next to it.  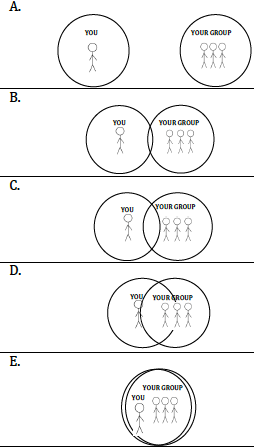 | 47. Think about yourself and your feelings for the OTHER GROUP (the one that does not share your colour). How close or distant do you feel to the other group? Choose from below ONE picture (A - E) that best represents how close or distant you feel to the other group. Put a cross in the ☐ next to it.  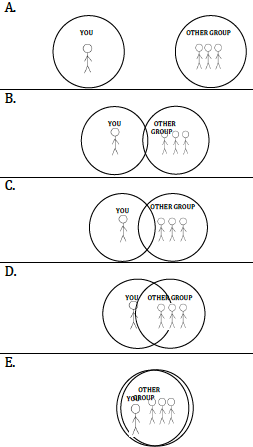 |
| *48. If we came back to do more activities another day, who would you choose to do them with? Pick ONE option below.*  ☐ 2 people from my group  ☐ 2 people from the other group  ☐ 1 person from my group and 1 person from the other group  ☐ Any 2 people - I don’t mind | |
