# Supplementary material for: Movement Synchrony Forges Social Bonds across Group Divides
Source: Front Psychol. 2016 May 27;7:782. doi: 10.3389/fpsyg.2016.00782 (PMC4882973; doi:10.3389/fpsyg.2016.00782)
Supplement: Supplementary file 2 [file Table2.DOCX]

| Table S2. *Analyses conducted to assess the suitability of the questionnaires for factor analysis* | | | |
| --- | --- | --- | --- |
|  | Kaiser-Meyer-Olkin Test  (threshold > .6) | Bartlett Test | Determinant values to check for multicollinearity  (threshold > .00001) |
| IB_pre-test_ | .62 | *X^2^* (3) = 43.61, *p* < .0001 | 0. 69344 |
| OB_pre-test_ | .73 | *X^2^* (3) = 176.25, *p* < .0001 | 0. 18672 |
| IB_post-test_ | .73 | *X^2^* (3) = 141.71, *p* < .0001 | 0. 34148 |
| OB_post-test_ | .74 | *X^2^* (3) = 155.73, *p* < .0001 | 0. 25762 |
| IB_long_ | .91 | *X^2^* (78) = 848.77, *p* < .0001 | 0. 00038 |
| OB_long_ | .94 | *X^2^* (55) = 896.53, *p* < .0001 | 0.00028 |
