# Supplementary material for: Movement Synchrony Forges Social Bonds across Group Divides
Source: Front Psychol. 2016 May 27;7:782. doi: 10.3389/fpsyg.2016.00782 (PMC4882973; doi:10.3389/fpsyg.2016.00782)
Supplement: Supplementary file 3 [file Table3.DOCX]

| Table S3. *Component loadings, communalities, eigenvalues of the components retained and the percentage of total variance explained based on principal component analysis with oblimin rotation for the short social bonding questionnaires* | | | | | |
| --- | --- | --- | --- | --- | --- |
|  | Loadings | | Communalities | | |
| Questionnaire Items | Pre-test | Post-test | Pre-test | | Post-test |
| I like my group \| the other group | .82 \| .93 | .86 \| .90 | .68 \| .86 | | .74 \| .81 |
| I would enjoy helping my group \| the other group | .80 \| .91 | .90 \| .89 | .64 \| .82 | | .81 \| .79 |
| My group \| The other group is cool | .67 \| .88 | .89 \| .90 | .44 \| .77 | | .80 \| .80 |
|  | Questionnaire characteristics | | | | |
| Questionnaire Items | Pre-test | | | Post-test | |
| Eigenvalues (in-group \| out-group) | 1.76 \| 2.45 | | | 2.35 \| 2.41 | |
| Percentage of total variance explained  (in-group \| out-group) | 59 \| 82 | | | 78 \| 80 | |
| Cronbach’s alpha for internal consistency  (in-group \| out-group) | .64 \| .89 | | | .85 \| .88 | |
