# Supplementary material for: Movement Synchrony Forges Social Bonds across Group Divides
Source: Front Psychol. 2016 May 27;7:782. doi: 10.3389/fpsyg.2016.00782 (PMC4882973; doi:10.3389/fpsyg.2016.00782)
Supplement: Supplementary file 4 [file Table4.DOCX]

| Table S4. *Component loadings, communalities, eigenvalues of the components retained and the percentage of total variance explained based on principal component analysis with oblimin rotation for the long social bonding questionnaires. Items with a “*−“ sign had *loadings < .4 and hence, were dropped from further analyses of the respective questionnaire.* | | |
| --- | --- | --- |
| Questionnaire Items | Loadings | Communalities |
| I feel connected to my group \| the other group | .73 \| .86 | .53 \| .74 |
| I feel on the same team with my group \| the other group | .76 \| .82 | .59 \| .68 |
| I feel good about my group \| the other group | .73 \| .85 | .53 \| .72 |
| I think I am similar to my group \| the other group in general | .73 \| .81 | .53 \| .66 |
| I share the same goals as my group \| the other group | .69 \| .74 | .47 \| .54 |
| I feel bad about my group \| the other group | -.72 \| − | .52\| − |
| I work well together with my group \| the other group | .79 \| .84 | .62 \| .70 |
| I am united with my group \| the other group | .73 \| .78 | .53 \| .61 |
| I don't like my group \| the other group | -.69 \| − | .48 \| − |
| I would stick together with my group \| the other group during challenges | .78 \| .85 | .60 \| .72 |
| I like my group \| the other group | .81 \| .82 | .66 \| .67 |
| I would enjoy helping my group \| the other group | .94 \| .84 | .70 \| .71 |
| My group \| the other group is cool | .80 \| .81 | .64 \| .65 |
| Eigenvalues (in-group \| out-group) | 7.39 \| 7.40 | |
| Percentage of total variance explained  (in-group \| out-group) | 57 \| 67 | |
| Cronbach’s alpha for internal consistency  (in-group \| out-group) | .82 \| .80 | |
