# Supplementary material for: Movement Synchrony Forges Social Bonds across Group Divides
Source: Front Psychol. 2016 May 27;7:782. doi: 10.3389/fpsyg.2016.00782 (PMC4882973; doi:10.3389/fpsyg.2016.00782)
Supplement: Supplementary file 5 [file Table5.DOCX]

| Table S5. *Frequencies (n) of children’s Island Game responses by factors sex, age, movement set and tempo and chi-square test results.* | | | | | |
| --- | --- | --- | --- | --- | --- |
|  |  | Own Group Island | Middle island | Other Group Island | *X^2^*  results |
| Sex | Male | 25 | 17 | 7 | *X^2^* = 4.92,  *p* = .09 |
|  | Female | 24 | 27 | 2 |  |
| Age | 7-year-olds | 5 | 3 | 1 | *X^2^* = 5.25,  *p* = .73 |
|  | 8-year-olds | 17 | 19 | 3 |  |
|  | 9-year-olds | 15 | 12 | 3 |  |
|  | 10-year-olds | 9 | 7 | 0 |  |
|  | 11-year-olds | 3 | 3 | 2 |  |
| Movement Set | Set 1 | 23 | 25 | 3 | *X^2^* = 2.00,  *p* = .37 |
|  | Set 2 | 26 | 19 | 6 |  |
| Tempo | 585ms | 23 | 24 | 4 | *X^2^* = 0.66,  *p* = .72 |
|  | 555ms | 26 | 20 | 5 |  |
