# Supplementary material for: Movement Synchrony Forges Social Bonds across Group Divides
Source: Front Psychol. 2016 May 27;7:782. doi: 10.3389/fpsyg.2016.00782 (PMC4882973; doi:10.3389/fpsyg.2016.00782)
Supplement: Supplementary file 7 [file Image1.PDF]

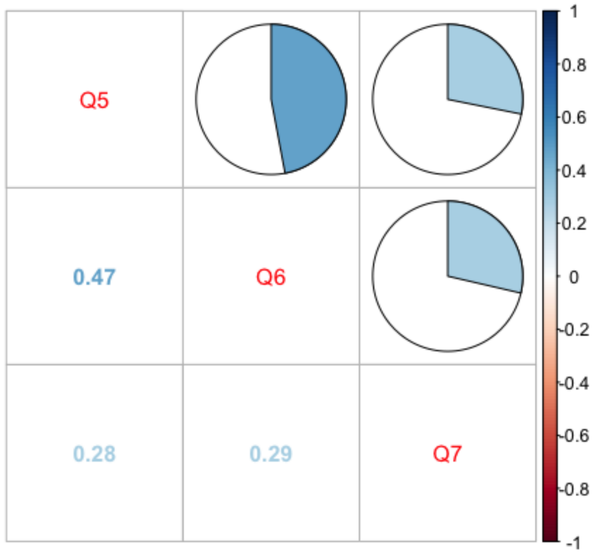

S1a. Pre-test in-group questionnaire

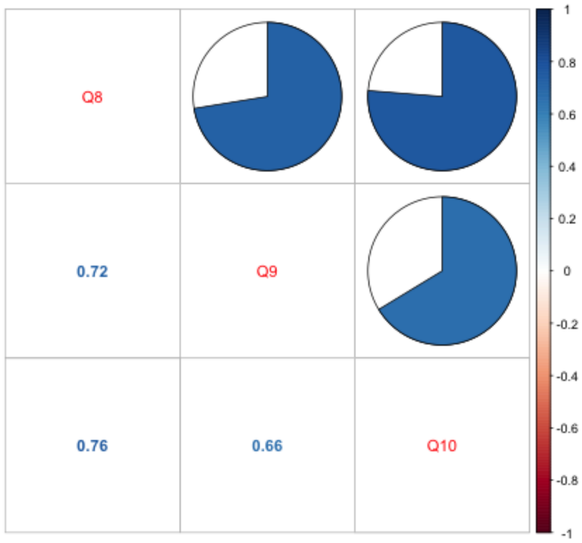

S1b. Pre-test out-group questionnaire

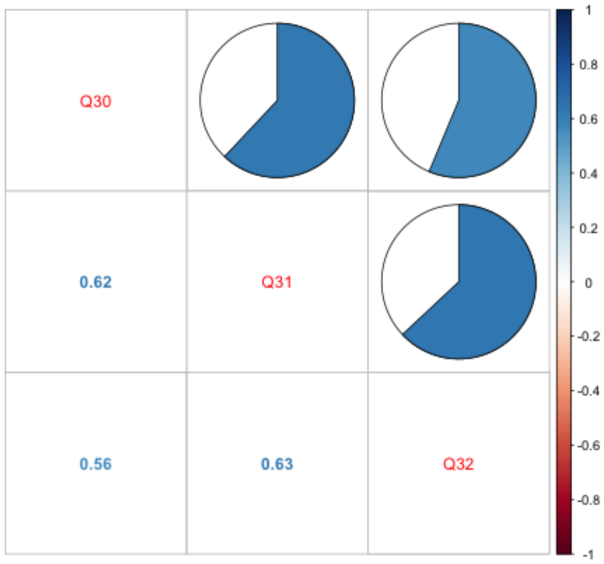

S1c. Post-test in-group questionnaire

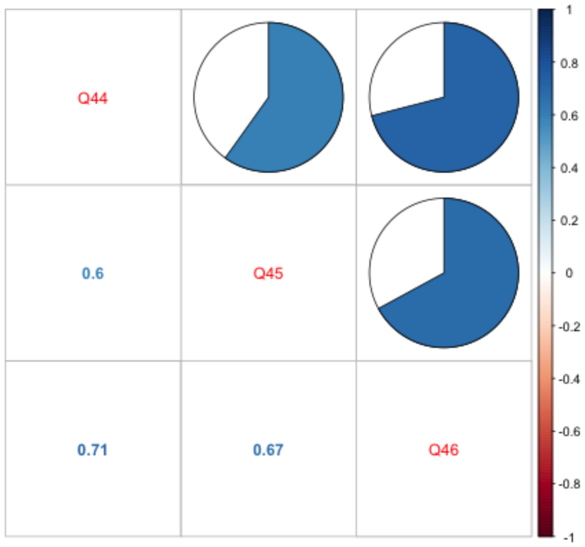

S1d. Post-test out-group questionnaire

Figure S1. Inter-item correlation matrices for the IB and OB questionnaires; all items were retained as their inter-item with correlations were between .3 and .9.
