# Supplementary material for: Movement Synchrony Forges Social Bonds across Group Divides
Source: Front Psychol. 2016 May 27;7:782. doi: 10.3389/fpsyg.2016.00782 (PMC4882973; doi:10.3389/fpsyg.2016.00782)
Supplement: Supplementary file 8 [file Image2.PDF]

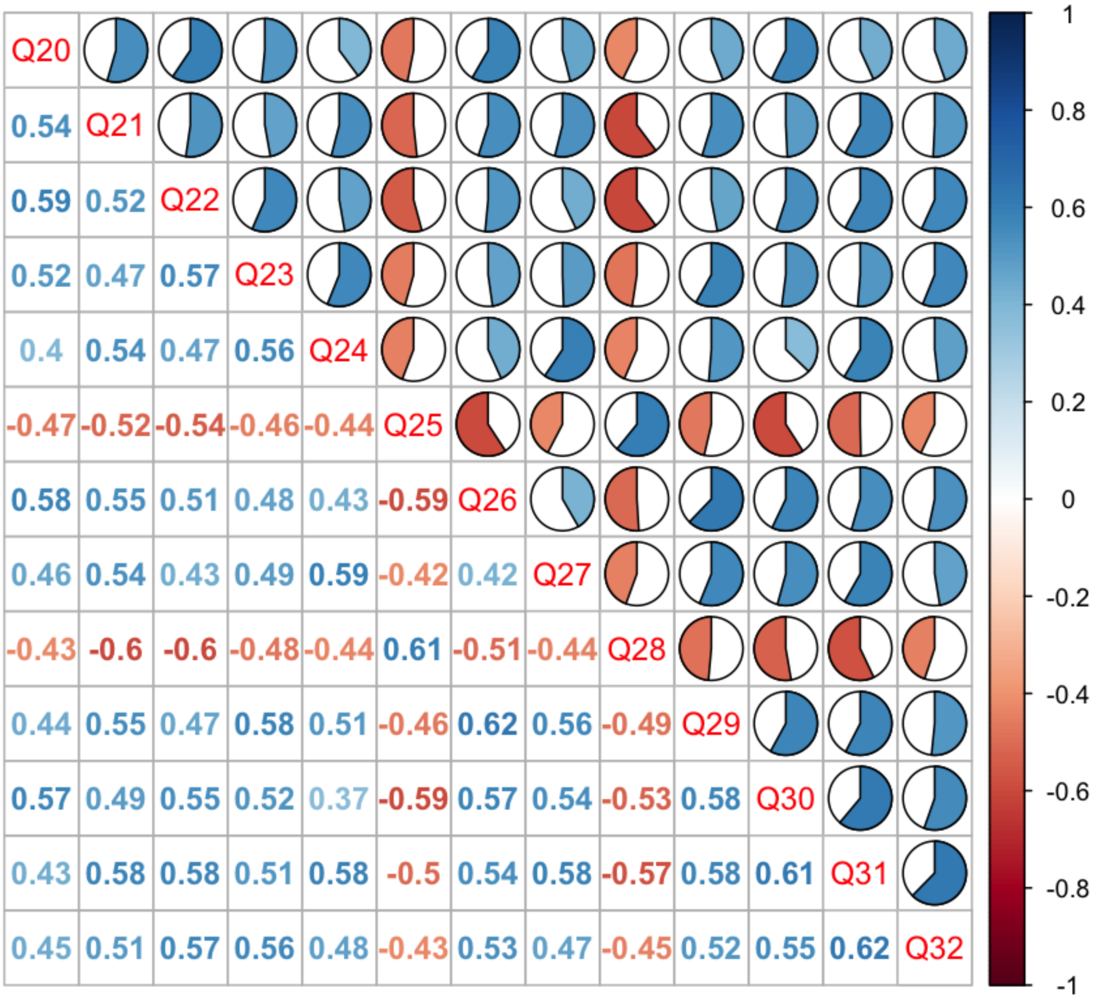

Figure S2. Inter-item correlation matrix for the IB_{long} questionnaire; all items were retained as their inter-item with correlations were between .3 and .9.
