# Supplementary material for: Movement Synchrony Forges Social Bonds across Group Divides
Source: Front Psychol. 2016 May 27;7:782. doi: 10.3389/fpsyg.2016.00782 (PMC4882973; doi:10.3389/fpsyg.2016.00782)
Supplement: Supplementary file 9 [file Image3.PDF]

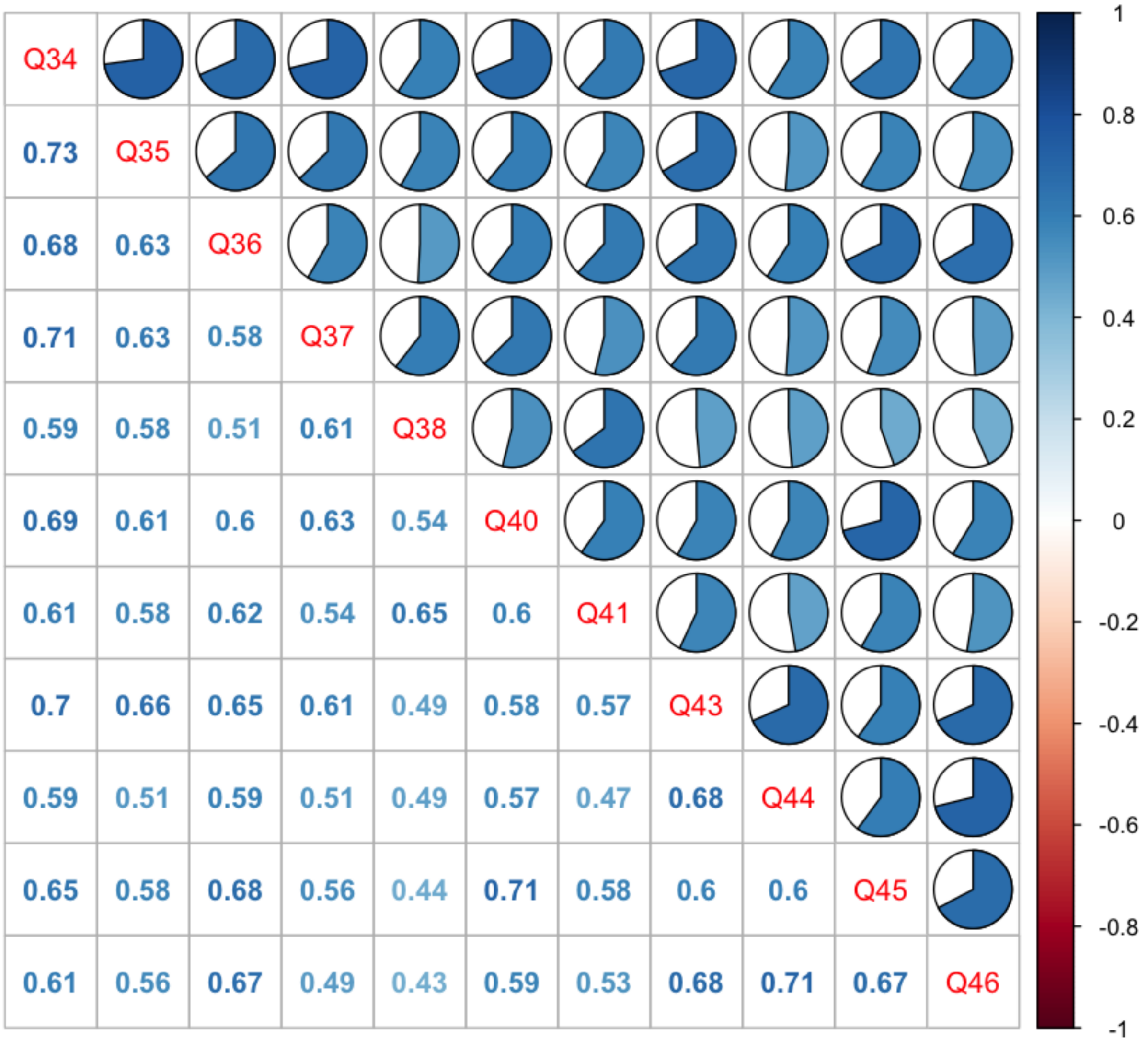

Figure S3. Reproduced inter-item correlation matrix for the OB_{long} questionnaire after items Q39 and Q42 were dropped due to having inter-item correlations < .3 and loadings < .4.
