# Supplementary material for: Movement Synchrony Forges Social Bonds across Group Divides
Source: Front Psychol. 2016 May 27;7:782. doi: 10.3389/fpsyg.2016.00782 (PMC4882973; doi:10.3389/fpsyg.2016.00782)
Supplement: Supplementary file 10 [file Image4.PDF]

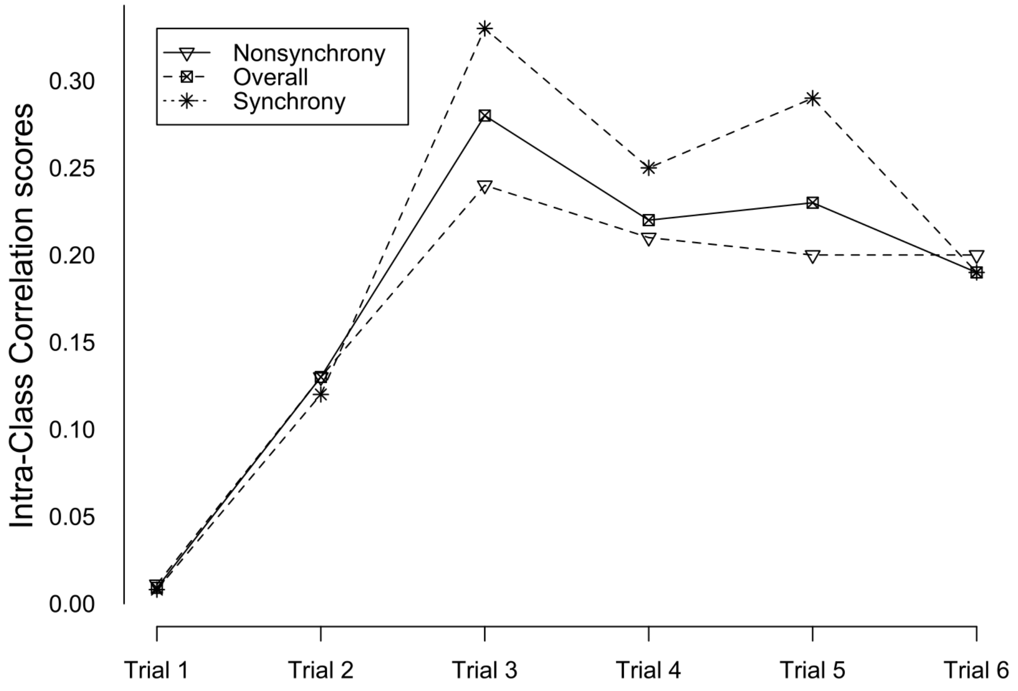

Figure S4. Intra-Class Correlations (ICC) by Island Game trial (1-6) for sessions in the synchrony condition, non-synchrony condition and for all sessions regardless of the c ondition
